# Single Molecule Experiments Challenge the Strict Wave-Particle Dualism of Light

**DOI:** 10.3390/ijms11010304

**Published:** 2010-01-21

**Authors:** Karl Otto Greulich

**Affiliations:** Fritz Lipmann Institute Beutenbergstr, 11 D 07745 Jena, Germany; E-Mail: kog@fli-leibniz.de; Tel.: +49-3641-656400; Fax: +49-3641-656410

**Keywords:** photon, wave particle dualism, single molecules, double slit experiment

## Abstract

Single molecule techniques improve our understanding of the photon and light. If the single photon double slit experiment is performed at the “single photon limit” of a multi-atom light source, faint light pulses with more than one photon hamper the interpretation. Single molecules, quantum dots or defect centres in crystals should be used as light source. “Single photon detectors” do not meet their promise—only “photon number resolving single photon detectors” do so. Particularly, the accumulation time argument, the only safe basis for the postulate of a strictly particle like photon, has so far not yet been verified.

## 1. Introduction

The particle aspect of light appears to be an undisputable fact. However, in the context of the photon, the definition of the term “particle” is not as straightforward as one might believe, with consequences for the meaning of the wave-particle dualism of light. Sometimes, the field quantization of light is interpreted as particle character, assuming delocalized quanta as photons. Experiments proving quantization, for example the Compton-effect or absorption of single light quanta by single atoms or molecules in a cavity do not strictly require that the photon be a particle in the common sense view. Certainly, the early pioneers of the wave particle dualism had a photon in mind which was related more strictly to this common sense view of a particle. Therefore the “accumulation time argument” was coined, which requires that the spatial dimensions of the photon be much smaller than the absorbing atom or molecule and that its whole energy content be concentrated in this limited volume. Otherwise the extremely short interaction time of a few femtoseconds would make it impossible that a single atom or molecule can absorb a single, freely travelling photon. It is this strict particle view, and not the fact of quantization of the light field, which is now challenged by single molecule experiments. The fact that light can be described as a quantized field is not disputed. For some, this distinction might appear simply as philosophical fine-tuning, but scientific rigor requires that a clear distinction is made between a strict particle and a more delocalized light quantum. It does make a difference if light is a beam of particles in the sense, which probably Newton (and even today most in the scientific public) had in mind, or a quantized electromagnetic field consisting of delocalized photons. The accumulation time argument (see for example [1]) and detailed properties of light generation and detection have to be taken into account. The classical discussions for example between Bohr and Einstein on particle properties of the photon have always tacitly assumed that isolated individual photons can be safely produced and safely detected. Feynman and many others assume that a click in a detector safely would count a single photon [2]. A classical experiment of Grangier *et al.* is thought to have shown that the photon is indivisible [3]. A re-examination of corresponding experiments shows that both Feynman’s and Grangier/Aspect’s views are not fully tenable. Regarding the claim that the photon is a particle no experimental proof exists, which undoubtedly proves this assumption. On the contrary, single molecule experiments rather indicate that the accumulation time argument has so far not yet been experimentally satisfied and that therefore there is no justification to postulate a true particle-like photon.

## 2. Results and Discussion

### 2.1. The Double Slit Experiment with a Single, Particle Like Photon is More Difficult than Einstein, Bohr, Feynman and Others Believed

*Gedankenexperiments* on the double slit experiment have provided the fundaments for believing that light behaves either as a beam of particles or as a wave, depending on the type of observation to be made. There are no indications in literature that the pioneers of our present model of light had a particle in mind, which would disagree with Newton’s photon particle and with the common sense view of a particle. However, the pioneers had to make assumptions on the function of light sources and detectors, which appeared to be reasonable in their time, but almost a century later turned out to be untenable. Single molecule experiments reveal a significant problem in the arguments supporting the strict particle property and thus the strict wave particle dualism of light. While the wave property of light is documented by Young’s double slit experiment, its strict particle property [4] is on shaky ground. The granularity of light, which Einstein had detected already in his seminal 1905 paper [5], does not necessarily require particles in the common sense meaning of the word.

In the classical double slit experiment (or corresponding modifications thereof) billions of photons at a time fall on the two slits and finally generate an interference pattern which reveals the wave property of light. In versions of the double slit experiment required to additionally reveal the particle property of light, exactly one isolated photon at a time is thought to be present in the apparatus. The essential claim of true single photon double slit experiments is that, when many of such single photons have passed the double slit, one will not see the classically expected result, namely intensity maxima directly behind the two slits, but the same interference pattern as in the classical double slit experiment. Since, on the one hand, single photons are thought to have been fed into the double slit and, on the other hand interference is a hallmark of waves, this experiment has coined our present notion of a wave-particle dualism of light.

A typical experimental realization of this experiment is as follows: the light source is highly attenuated and the detector(s) are tuned down so far that they reveal only one click per time interval. This is typically interpreted as single photons (single photon quantum states, Fock n = 1 states) passing the double slit. Many experimenters are careful enough to speak of “single photon pulses” and not of single photons. In reference [6], published in 2007, doubts have come up that earlier experiments indeed have used single photon quantum states. Thus one needs to think about whether theoretical discussions of these experiments including the famous ones between Albert Einstein and Niels Bohr may remain pure *Gedankenexperiments*. In the following, first on purely statistical arguments, then with the results of a recent single molecule experiment, it is shown that arguments in favour of a strict particle property indeed stand experimentally on brittle ground. In a second step it will be additionally shown, again based on single molecule arguments, that the strongest argument of physics in favour of a strict particle property of light, the accumulation time argument, is so far not covered by experimental facts. Some of these points have already been discussed in [7] (http://www.fli-leibniz.de/www_kog/, then click the Φ), in the context of further information which also explains in detail, why short wave radiation is perceived as a beam of particles and long wave radiation as wave-like.

### 2.2. The Difficulty to Generate, by Attenuation or Dilution, Single Photons from Multi-atom Light Sources

Understanding numerical details on generating single photons from multi-atom light sources is absolutely mandatory. However, these details are rarely discussed in experiments crucial for our understanding of the nature of light. Some “dry” calculations are required, which are essentially always omitted in discussions of experiments on the nature of light. For the following it is useful to recall that the small energy portion of 1 nanojoule represents still a very large number of photons, namely 2.5 billion in the case of green photons of 500 nm wavelength. Assume that a 0.1 Watt Titan Sapphire laser at 500 nm with a pulse-duration of 100 femtoseconds is used. At a typical pulse repetition rate of 100 MHz, each one has an energy of 1 nanojoule = 2.5 × 10^9^ photons. If this beam has a diameter of a millimeter and the apparatus has an entrance slit of a micrometer, 1 ppm (=2,500 photons per pulse) are admitted to the apparatus. This corresponds to a very sharp spatial filtering. For simplicity assume, certainly much too optimistic, that the error of the just described process is zero. The 2,500 photons have now to be attenuated to 1 photon by removing 2,499 photons. This attenuation probably has a Gaussian error of √ 2499 = 49.99. The result is on the average 1 photon over many, often empty, pulses with a Poisson-distribution among the individual pulses. The critical point is that the standard deviation is 49.99 photons. Thus, concluding that one works in the “single photon limit” when one has attenuated to an average of 1 photon per time unit is risky. Therefore, experiments on quantum information transfer and non locality working in the single photon limit do probably not provide the physical information they are often thought to provide.

### 2.3. Single Molecule Experiments Show That Even a Two Molecule Light Source Generates Bunching Light

**Figure 2.**
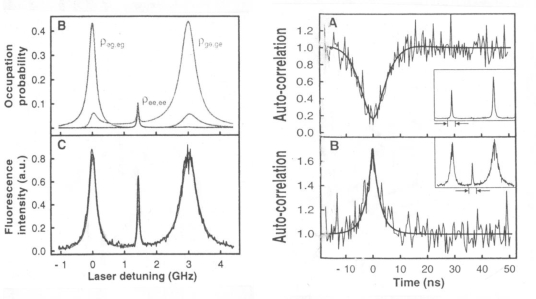
Left panel: B: Occupation probability and C: fluorescence intensity at slight de-tuning of a laser source for two emitters at a distance of 12 nm. Right panel: A: Antibunching when the laser is tuned either to molecule 1 or to molecule 2. B: Bunching when it is tuned to the cross peak. Reproduced from [8].

Even if the statistical problems described so far could be solved, another, so far unrecognized complication emerges: bunching of photons even from a two molecule source. Two terrylene molecules are embedded in a *para*-terphenyl crystal at a distance of 12 nm [8]. Due to slight differences in their microenvironment, their excited state occupation probabilities and the resulting fluorescence peaks are slightly different (approx. 3 GHz), so that the excitation source could be tuned to generate fluorescence either from molecule 1 or from molecule 2. After tuning the excitation source for molecule 1, it behaves like an isolated single molecule, *i.e.*, it emits anti-bunching light as expected. Correspondingly, molecule 2 behaves “correctly”. However, the optical properties of this molecule-pair are not just the sum of each of the parts, but reveal a cross peak at half the wavelength between the absorption peaks of the individual molecules (Figure 2, left panels). If the excitation source is tuned to this cross-peak, a surprising effect occurs: the emitted light is bunching Figure 2, right panels). Bunching and the existence of a cross-peak indicate that both molecules co-operate and do no longer emit strictly independent photons. While in a two molecule source, and perhaps in a source consisting of a few molecules, it is still possible to tune an exciting laser to exactly one molecule and thus still may get single antibunching photons, this is, simply for practical reasons, not possible for a real multi-atom source with millions or even billions of emitters. This result of ref. [8] was explained via dipole-dipole coupling and was essentially confirmed in a completely different type of experiment [9]. As a consequence, the experimenter is only on the safe side in preparing single photons when one uses single, isolated atoms or molecules. The latter may reside for example on a microscope slide or in a quantum cavity.

### 2.4. The Accumulation Time Argument Has So Far Not Yet Been Safely Verified

Essentially the only argument in favour of a strict particle-like photon is the “accumulation time argument”. It says that the “flyby” time of an extended (non particle like) photon would be too short to allow absorption by a single atom or molecule [1]. In the past, it has always been assumed, without experimental proof, that a single atom or single molecule in free space can be excited by a single photon travelling freely through space. However, this experiment is still elusive and thus an experiment clearly in favour of a strictly particle like optical photon is still missing. So far, only multi-atom sources have been used for single molecule excitation. An intensity of 0.1 mW/cm^2^ indeed provides a sufficiently low average photon flow, but as discussed above, working in the single photon limit is not safe. Alternatively one can excite a single atom or molecule by a pulsed laser, but then one needs kilowatt peak powers, far from the one photon required. Note that the successful excitation of a single atom by a single photon in a cavity does not save the accumulation time argument, since in this case a longer interaction time than in “flyby” of a freely travelling photon can at least not be ruled out.

### 2.5. Even Theories Do Not Clearly Demand a Strictly Particle Like Photon

Richard Feynman is convinced “*that light is made of particles because we can take a very sensitive instrument that makes clicks*” [2]. In the next chapter it will be discussed that this reasoning is not tenable. Also, in his 2005 Nobel Prize lecture Roy Glauber states on Albert Einsteins idea to invoke a particle aspect of light: “*That was a suggestion that light itself might be discrete in nature*, *but hardly a conclusive one*” and on the idea that the Compton effect would prove the particle aspect of light: *“It became clear that the particle picture of light quanta*, *whatever were the dilemmas that accompanied it*, *was here to stay* [10]. Note that Glauber speaks of the “*particle picture of the quanta*”, obviously having in mind, that the term “quanta” does not automatically imply “particle”. Thus, experiments proving the quantized nature of light do not necessarily prove the particle aspect of the photon. This does occasionally not become clear in discussions of the photon, for example in [4]. Notions that discussing this difference might be “philosophical fine tuning” are probably not tenable. Both statements of Roy Glauber indicate that even from the viewpoint of the year 2005, the strict particle aspect of the photon does not stand on solid ground as it is often believed.

### 2.6. “Single Photon Detectors” Do Not Safely Count Single Photons

Occasionally it is suggested to use single photon detectors and to tune an experiment so far that even a multi-atom source provides single photons. This approach has its own pitfalls. A number of companies provide “single (optical-) photon detectors”. All of those, which have so far been asked, how these detectors were calibrated, had to concede that calibration has not been performed using a single molecule source but using attenuated pulses, with exactly the problems described in the previous chapters. Detectors based on a photoelectron multiplication process can indeed detect one photoelectron. They do, however, not register the number of photons required to generate this photoelectron. Thus, a single click in the detector, though it may indeed result from a single photon, may also result from a light pulse containing many photons. In other words, a click in the detector needs not to be caused by a single, particle like photon, as it is claimed in many discussions on experiments thought to prove the wave particle dualism of light. Real photon-number-resolving single photon detectors are required. The only class of optical photo detector which approaches that aim is that working on a calorimetric principle [11]. The need for this type of detector in experiments on the Bell inequalities has been pointed out by Khrennikov [12], Adenier [13] and in ref. [14]. Calorimetric detectors are very slow (microseconds) and have so far not yet been used in experiments on the nature of the photon and light.

### 2.7. The Indivisibility of the photon is not proven. Once giving it up, Classical Explanations Are Possible

An experiment which is often claimed to have proven indivisibility of the photon is the one of Grangier *et al.* [3] or similar work of the same group. This experiment was interpreted to show strong anti-correlation since a beam splitter did not divide the faint light pulses used. The paper claimed to be the first which really used single photon states and invalidated earlier experiments on single photon interference since the latter had not really used such single photons. However, in their 2007 paper [6] the same authors came to the conclusion that even ref. [3] and similar papers had not really used single photon states. By now single photon quantum states have not been safely used to prove their indivisibility. Until an unequivocal proof is presented, a divisible photon is equally possible.

Muthukrishnan and Roychoudhuri state: “the indivisibility of the photon thus remains an open question and one that we can use to probe the foundations of quantum electrodynamics” [15]. Once giving up the indivisible photon, interference, for example in a double slit experiment, can be classically explained by properties of beam splitters, Wollaston prisms and detectors in the experimental setup, even when true single photon quantum states are used [16]. In short, when a photon is divisible, its parts can travel two distinct paths. As long as they are divided the parts cannot be detected for energy reasons. However, when the parts are recombined, both parts either act at the atoms, molecules or band structures of the detector material in a constructive way to excite the detector or in a destructive way to cancel each other’s action. This gives an interference pattern exactly as if a wave would have travelled the two paths.

## 3. Conclusions

In conclusion, neither the strict particle nature of the photon nor its indivisibility has so far been safely proven. The assumptions of Einstein, Bohr and others have never been fully satisfied. The edifice of a strict wave particle dualism of light is still based rather on *Gedankenexperiments*. Only now, with the advent of robust single molecule light sources [17–19], quantum dots or for example colour centres in solid state bodies [20,21] the really suitable experiments are becoming possible. The experiment meeting best the necessary requirements is that of Jacques *et al.* [6], but even there the description of experimental details is not completely sufficient to eliminate all doubts [16]. While many experiments indicate the existence of photons as energy packages and thus confirm field quantization, not a single experiment is available which demands a strictly particle like photon.

## Figures and Tables

**Figure 1.**
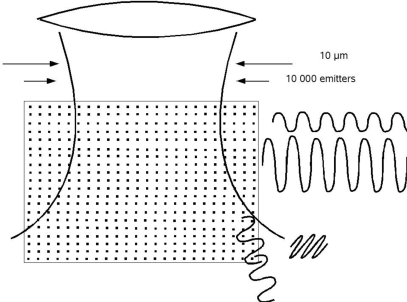
The geometry in a real multi-atom or molecules light source. Always billions of emitters are involved and cooperating bunches are emitted. Even a poor-quality source reveals some coherence (see also next section).
